# Epigenetic Changes Associated With Interleukin-10

**DOI:** 10.3389/fimmu.2020.01105

**Published:** 2020-06-04

**Authors:** Zhonghua Zheng, Gang Huang, Tong Gao, Tianyi Huang, Mengsha Zou, Yuhao Zou, Shiwei Duan

**Affiliations:** Medical Genetics Center, School of Medicine, Ningbo University, Ningbo, China

**Keywords:** DNA methylation, epigenetics, interleukin-10, immune inflammatory disease, histone modification, microRNA, lncRNA

## Abstract

IL-10 is a regulator of inflammation and immunosuppression. IL-10 regulates a variety of immune cells to limit and stop the inflammatory response, and thus plays an important role in autoimmune diseases, inflammatory diseases and cancer. IL-10 is closely related to epigenetic modification, in which changes in DNA methylation of IL-10 gene can affect mRNA and protein levels of IL-10. In addition, changes in histone modifications, especially histone acetylation, can also lead to abnormal expression of IL-10 mRNA. At the same time, a handful of IL-10 related microRNAs (miRNAs) are found to be aberrantly expressed in multiple diseases. Besides, long non-coding RNA (lncRNA) growth arrest specific transcript 5 (GAS5) also inhibits IL-10 expression. Here, we reviewed the epigenetic changes related to IL-10 in various diseases, as well as the regulation of IL-10 gene expression in various diseases by epigenetic modifications such as DNA methylation, histone modification, miRNA, and lncRNA.

## Introduction

As the primary member of the IL-10 cytokine family (1), IL-10 plays an important role in the regulation of the differentiation and proliferation of a variety of immune cells, such as T cells, B cells, natural killer cells, antigen presenting cells, mast cells, and granulocytes (2). The transcription mechanism of IL-10 is also very complicated. Foxp3, Nfil3, Ets1, and other transcription factors can modify the chromatin state of IL-10 by recruiting various effector proteins (3). The major biological function of IL-10 is to limit and stop the inflammatory response, which is pivotal in autoimmune diseases, inflammatory diseases and cancer (4, 5).

Epigenetics refers to modifications in the genome that do not alter the DNA sequence, including DNA methylation, histone modifications, non-coding RNA, etc. (6). Epigenetic modifications can respond to environmental stimuli by activating or inhibiting gene transcription (7). Recent studies have shown that epigenetic regulation of IL-10 expression can influence the progression of diseases such as Behçet's's disease and atherosclerosis (8, 9).

## IL-10 Structure

IL-10 is a regulator of inflammation and immunosuppression (10). Human IL-10 gene is 4,893 bp in length and includes 5 exons and 4 introns, which are located at 1q32.1, encoding genes on the minus strand. The human functional IL-10 protein is a dimer of 160 amino acids after removal of the 18 amino acid signal peptide (11, 12). Each strand of mouse IL-10 (mIL-10) consists of 157 amino acids and its amino acid sequence is ~75% identical to human IL-10 (13). There are some important single nucleotide polymorphisms in the IL-10, including −819T/C (rs1800871), −592A/C (rs1800872), and −1082G/A (rs1800896), etc. (14). These genetic variants can regulate IL-10 expression levels, thereby affecting the progression and severity of IL-10-related diseases (15, 16).

## DNA Methylation Modification Associated With IL-10

**Figure 1:**
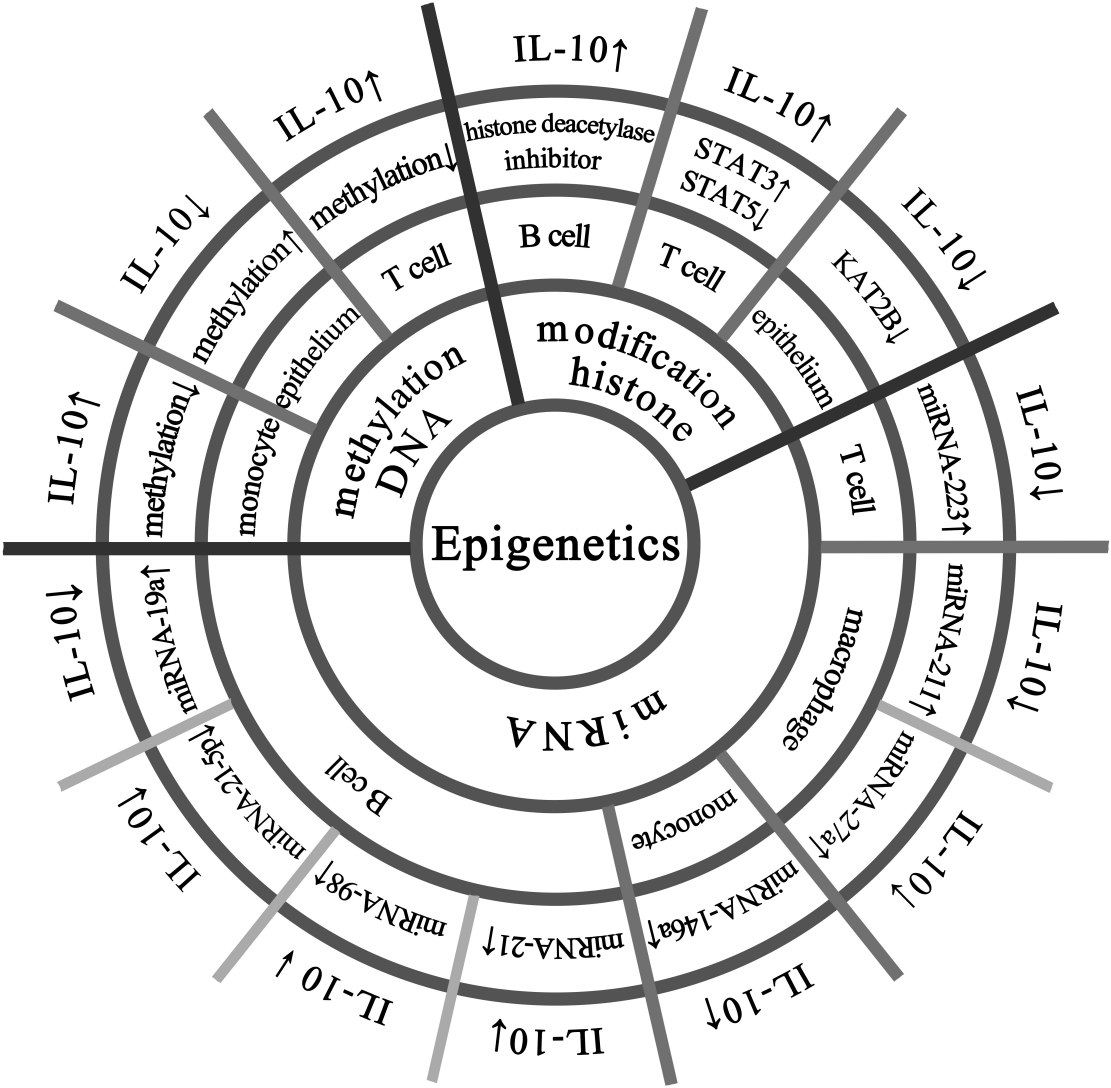
Epigenetic modifications that regulate IL-10 expression in multiple cells. The IL-10 promoter has transcriptional activity and hypomethylation in human peripheral blood mononuclear cells, while the IL-10 promoter is silenced in epithelial cells due to hypermethylation. There is an enhancer in the IL-10 gene intron in T cells, and its hypomethylation promotes IL-10 expression. B cells treated with histone deacetylase inhibitor and 5-aza can increase the expression of IL-10. In T cells, increased activation of STAT3 can lead to enhanced recruitment of regulatory regions and competitive replacement of STAT5, promoting IL-10 expression. In normal colonic epithelial cell line (NCM460), laccase acid inhibits KAT2B and reduces the transcriptional activity of KAT2B and H4K5ac on the IL-10 promoter, thereby significantly down-regulating the expression of IL-10. Overexpression of miRNA-146a in peripheral blood mononuclear cells increases IL-10 expression. Up-regulation of miRNA-19a in B cells reduces IL-10 expression, and the same up-regulation of miRNA-98 can also suppress IL-10 expression, and insufficient expression of miRNA-21-5p is one of the reasons for the decrease in IL-10 expression in B cells. Up-regulation of miRNA-21 inhibits the differentiation of IL-10 + Breg cells and promotes the expression of miRNA-223 in autoimmune T cells, resulting in reduced IL-10 production. In macrophages, the relative expression of miRNA-211 is abnormally upregulated, accompanied by a decrease in secreted IL-10. miRNA−27a can enhance the antibacterial activity of macrophages and inhibit the expression of IL-10.

The IL-10 protein is ubiquitously expressed in various tissues of mice, and the methylation level of the IL-10 gene is not high and specific in each tissue (17). As shown in Figure 1, DNA methylation regulates IL-10 expression in different cells. Larsson et al. have shown that the methylation status of the human IL-10 promoter is closely related to the transcriptional activity of IL-10 (18). Specially, in human peripheral blood mononuclear cells, the hypomethylation of the IL-10 promoter corresponds to higher IL-10 expression; in the epithelial cells, the IL-10 promoter shows hypermethylation to silence IL-10 expression (18). Hedrich et al. have shown that the IL-10 gene intron has an enhancer, and the decrease in DNA methylation of the enhancer in T cells of systemic lupus erythematosus (SLE) patients can increase the recruitment of STAT transcription factors and promote IL-10 expression (19). Besides, Yu et al. have found that arginine can induce hypomethylation of IL-10 promoter DNA, thereby regulating IL-10 production in neonatal Treg cells (20). The methylation status of the IL-10 locus promoter in Th1 cells can be reversibly regulated, which may be used to rapidly regulate the transcription level of IL-10 gene (21). Lorente-Sorolla et al. found that increased IL-10 levels were associated with changes in IL-10 methylation in circulating monocytes in patients with sepsis (22). At present, DNA methylation modification has been recognized as a key regulatory mechanism for IL-10 gene expression (8).

As shown in Table 1, IL-10 DNA methylation levels are associated with many diseases. As the severity of liver failure increases, the level of IL-10 in serum of patients with hepatitis increases, and the decrease in methylation of IL-10 promoter plays an important role in IL-10 gene activation (23). IL-10 intron methylation levels were higher in asthmatic patients than in non-asthmatic patients, and changes in IL-10 different methylation regions were associated with the exposure risks of CO, NO_2_, and PM2.5 (24). In addition, the IL-10 gene is hypermethylated in periodontal tissues (27). Hypermethylation of CpG islands in the IL-10 promoter region may be involved in the development of rheumatoid arthritis (RA) (28). In addition, IL-10 gene methylation levels were decreased in SLE CD4+ T cells compared with healthy controls, and negatively correlated with IL-10 mRNA expression (30). The hypomethylated IL-10 gene is associated with higher SLE disease activity, and serum IL-10 levels are also reduced in RA patients. Therefore, IL-10 hypomethylation may provide a potential epigenetic marker for clinical prediction of autoimmune diseases (29). A study found that increased FOXP3-TSDR methylation levels were significantly associated with the severity of atherosclerosis and the changes in IL-10 concentration (31).

**Table 1:** Relationship between IL-10 DNA methylation and various diseases.

**Disease**	**Samples (*n*)**	**Method**	**Methylation level**	**References**
ACLF	ACLF (25), CHB (25), Healthy controls (10)	MSP	Hypermethylation	(23)
Asthma	Students of Fresno Unified School (188)	Bisulfite pyrosequencing	Hypermethylation	(24)
BD	Peripheral blood of patients (51), Healthy controls (63)	MeDIP-qPCR	Hypermethylation	(8)
Breast cancer	Breast cancer tissues (72), Benign tissues (31), Normal tissues (30)	Bisulfite sequencing	Hypomethylation	(25)
CLL	CLL (27, CSU cohort), CLL (36, DKFZ cohort)	BSP	Hypomethylation	(26)
Periodontal disease	Gingival tissue (34)	MSP	Hypermethylation	(27)
RA	RA patient (34), Healthy controls (30)	MSP	Hypermethylation	(28)
SLE	SLE (66), RA (12), Healthy controls (102)	Whole genome methylation array	Hypomethylation	(29)
SLE	SLE (30), Healthy controls (26)	Bisulfite Sequencing	Hypomethylation	(30)

*ACLF, acute on chronic liver failure; BD, Behçet's disease; CLL, chronic lymphocytic leukemia; CSU, Cancer Sciences Unit in Southampton; DKFZ, German Cancer Research; RA, rheumatoid arthritis; SLE, systemic lupus erythematosus*.

IL-10 promoters tend to be hypomethylated in different cancer types (32). Lima et al. showed that there were 37 CpG sites with methylation differences between esophageal squamous cell carcinoma (ESCC) and adjacent non-tumor tissues, and these sites were associated with IL-10-related pathways including anti-inflammatory signaling pathways, and cellular communication pathways, etc. (33). Alipour et al. collected blood samples from 51 Behçet's disease patients and 63 healthy controls, evaluated promoter methylation levels by MeDIP-qPCR, and subsequently assessed IL-10 expression by Real-time PCR (8). And their study found that the expression level of IL-10 gene was significantly decreased in blood samples from patients with Behçet's disease, mainly due to the high methylation of the IL-10 promoter region resulting in decreased mRNA expression levels (8). In addition, IL-10 promoter hypomethylation and IL-10 overexpression were observed in breast cancer tumor tissues compared to normal breast tissue and benign breast tissue (25). Drennan et al. showed that IL-10 gene hypomethylation is a prominent feature of chronic lymphocytic leukemia (26). At the same time, Tonon et al. found that IL-10 demethylation corresponds to IL-10 expression in mouse and human B cell-associated cancers (34). Epigenetic changes, such as abnormal DNA methylation of IL-10, are also closely associated with the development and progression of colorectal cancer (35). The main thrust of our review is to introduce the epigenetics of IL-10, so we have not introduced many studies on IL-10 polymorphism.

## IL-10 DNA Methylation Levels May Be an Important Feature of the Disease

The level of IL-10 DNA methylation is affected by a variety of factors, which presents different levels of modification in different diseases and different tissues. The severity of the disease affects the methylation level of IL-10. Hepatitis patients with increased liver failure will lead to IL-10 promoter methylation and IL-10 gene inactivation. In addition, IL-10 methylation increased significantly during short and long-term exposure to high levels of CO, NO_2_, and PM2.5. In cancer, the IL-10 promoter is often hypomethylated. In summary, the level of IL-10 methylation modification may become a marker for diagnosis and prognosis of disease.

## Histone Modifications Associated With IL-10

**Figure 2:**
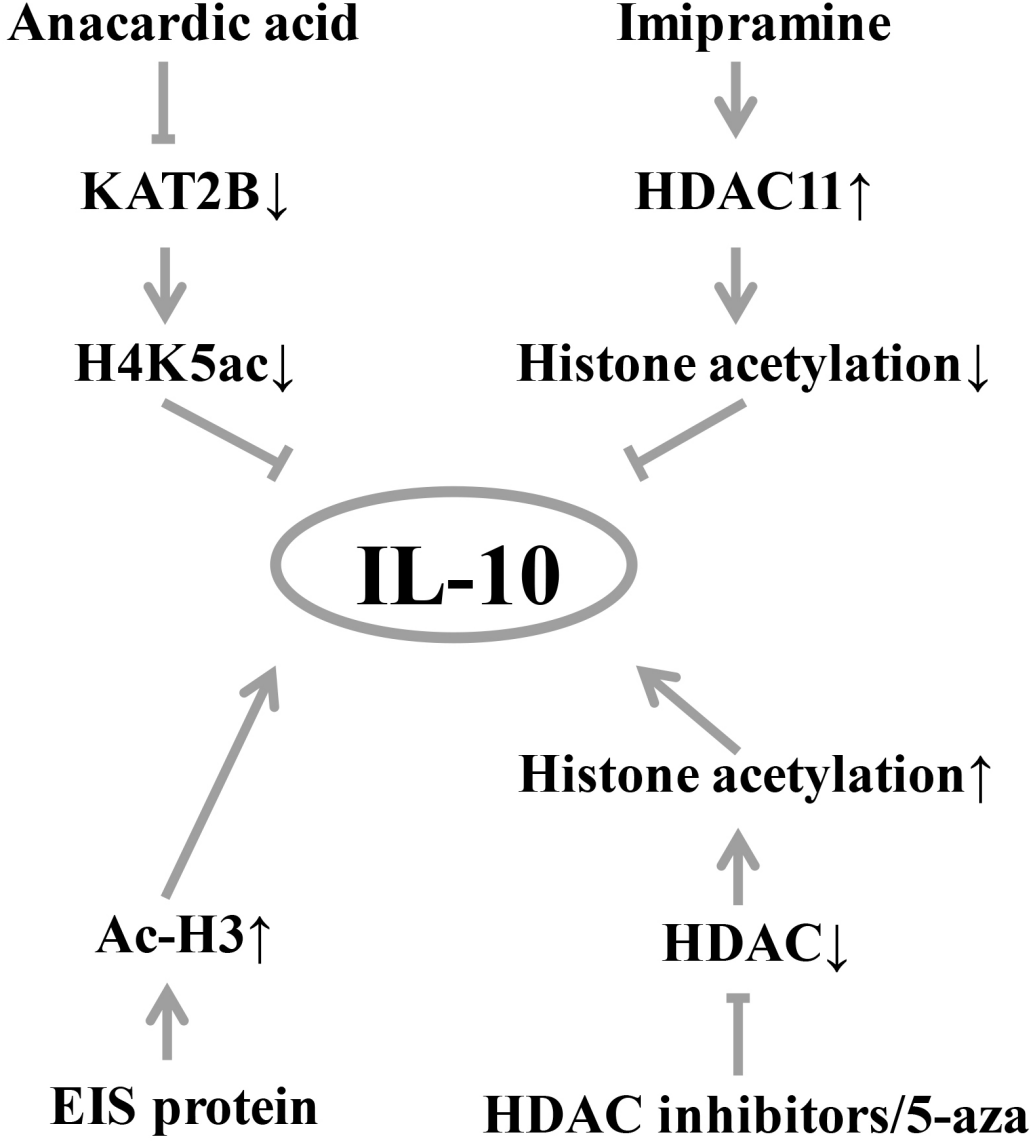
Diagram of IL-10 and histone modification. Histone acetylation induces the production of analgesic factors such as IL-10. EIS protein can significantly increase the level of acetylation of histone H3 (Ac-H3), thereby increasing the expression of IL-10. Histone acetylase inhibitors and 5-aza treatment increased IL-10 mRNA expression. Inhibition of histone deacetylase by histone deacetylase inhibitors, histone acetylation induces spinal cord production of analgesic factors including IL-10. Conversely, the inhibition of anacardic acid down-regulated KAT2B, thereby reducing the occupancy of KAT2B and H4K5ac by the IL-10 promoter, resulting in down-regulation of IL-10 expression by transcriptional silencing. Similarly, imipramine upregulates histone deacetylase 11, which inhibits the acetylation of the IL-10 promoter resulting in a decrease in IL-10 production. KAT2B, lysine acetyltransferase 2B; H4K5ac, histone H4 lysine 5 acetylation; HDAC, histone deacetylase; Ac-H3, acetylation level of histone H3; EIS, enhanced intracellular survival.

Epigenetic modifications of DNA-binding proteins, such as histone acetylation, are also important factors influencing gene expression (36). A handful of studies have found that histone modifications can affect IL-10 level (Figure 2). Treatment of B cells with histone deacetylase inhibitor and 5-aza-2′-deoxycytidine (5-aza) increases IL-10 mRNA transcription (18). In a mouse experiment, histone acetylation induces spinal cord production of analgesic factors including IL-10 (37, 38). In T cells from SLE patients, increased expression of STAT3 promotes its binding to the regulatory region of IL-10 and competitively replaces the binding of STAT5 to the regulatory region of IL-10, which subsequently promotes IL-10 gene expression (19). In the normal colonic epithelial cell line (NCM460) (Figure 1), anacardic acid down-regulates the KAT2B gene, reducing the transcriptional activity of KAT2B and H4K5ac on the IL-10 promoter, thereby significantly downregulating IL-10 expression (39). In addition, imipramine upregulates histone deacetylase 11, which inhibits the binding of the acetylated histone of the IL-10 gene promoter and reduces IL-10 expression (40). Duan et al. found that enhanced intracellular survival (EIS) protein significantly increased the acetylation level of histone H3 (Ac-H3), which in turn increased the binding of Ac-H3 to the SP1 and STAT3 regions of the human IL-10 gene promoter, thereby promoting the expression of IL-10 (41).

## Important Role for Histone Modification of IL-10

As shown in Figure 2, treatment of B cells with histone deacetylase inhibitor and 5-aza can increase the expression of IL-10 mRNA, and the inhibition of KAT2B by ursolic acid can significantly down-regulate the expression of IL-10. In addition, the up-regulation of imipramine can reduce IL-10 production.

## IL-10-Associated miRNA

**Figure 3:**
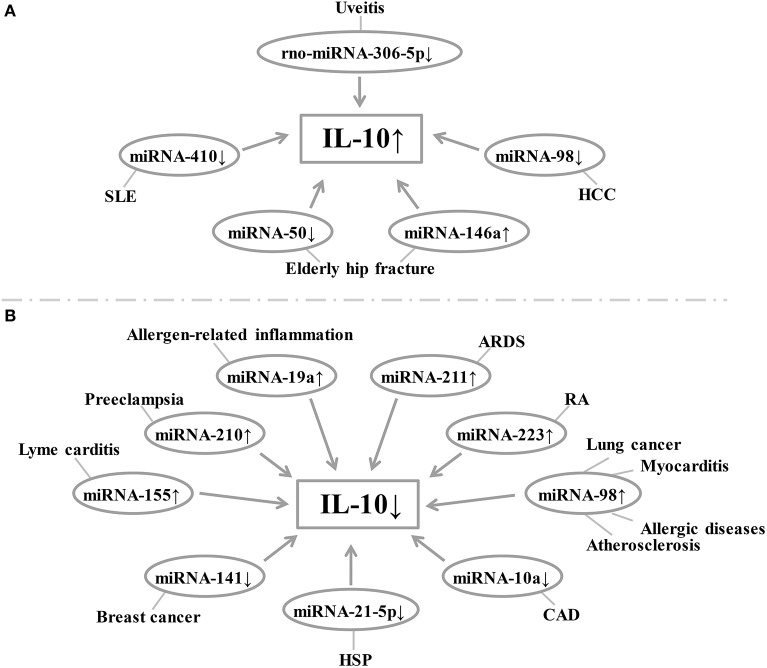
**(A,B)** Relationship between miRNA expression and IL-10 levels in each disease. Changes in the level of expression of miRNAs in each disease result in changes in IL-10 levels. SLE, systemic lupus erythematosus; HCC, hepatocellular carcinoma; ARDS, acute respiratory distress syndrome; RA, rheumatoid arthritis; CAD, coronary artery disease; HSP, Henoch-Schonlein purpura.

The miRNAs are a class of non-coding RNA molecules ranging in length from 18 to 24 nucleotides (42). In peripheral blood mononuclear cells, IL-10 expression is inversely correlated with the level of miRNA-98-5p (43). Overexpression of miRNA-146a increased IL-10 expression in peripheral blood mononuclear cells [Figure 1; (44)]. In breast cancer cells, down-regulation of miRNA-141 inhibits IL-10 and leads to up-regulation of COX-2, PGE-2, and TNF-α expression (45). Increased IL-10 release after hip fracture can lead to significant systemic inflammatory response and acute lung injury (ALI) after hip fracture, and increased IL-10 release is significantly associated with up-regulation of miR-146a and down-regulation of miRNA-150 (46). Besides, miRNA-10a is found to be positively correlated with serum IL-10 concentration and plays an important role in atherosclerotic inflammation (47). miRNA-155 has been shown to play a role in immune activation and inflammatory responses and is inhibited by IL-10 (48, 49). Similarly, IL-10 has been found to inhibit TLR4-induced miRNA-155 expression (50). The antagonism between IL-10 and miRNA-155 is necessary to balance host defense and immune activation *in vivo*, and this balance is particularly important in inhibiting Lyme myocarditis (51). Besides, TNF-α, IFN-γ, and IL-4 inhibit IL-10 expression in B cells by up-regulating miRNA-19a expression (52, 53). Liu et al. found that miRNA-19a levels were higher in B cells of sensitized mice in inducing intestinal allergy-related inflammation, thereby inhibiting IL-10 expression in B cells (54). The miRNA-210 expression was significantly elevated in placenta of preeclampsia (PE) patients, while IL-10 levels were significantly lower than normal pregnant women (55). Luo et al. found that the expression of IL-10 was positively correlated with the expression of miRNA-21-5p in B cells of patients with allergic purpura (HSP) (56). In BALF-derived macrophages from ARDS rats, the relative expression of rno-miRNA-211 was up-regulated, accompanied by a decrease in secretory IL-10 (57). Lu et al. have shown that increased expression of miRNA-223 in T cells of RA patients leads to a decrease in insulin-like growth factor-1 mediated IL-10 production, leading to an imbalance between pro-inflammatory cytokines and anti-inflammatory cytokines (58). Sun et al. reported that rno-miRNA-30b-5p is down-regulated in the spleen, lymph nodes, and eye tissues of experimental autoimmune uveitis rats, while down-regulation of rno-miRNA-30b-5p may be regulated by IL-10 and TLR4 levels affect the proportion of IL-10 positive cells in the cell population, thereby inhibiting the development of uveitis. rno-miRNA-30b-5p mimics can reduce the expression of IL-10 and TLR4 genes and proteins, thereby affecting the pathogenesis of uveitis. Therefore, rno-miRNA-30b-5p may be a new therapeutic target for uveitis (59). In addition, studies have shown that miRNA-410 is a key regulator of the pathogenesis of SLE, and it regulates IL-10 expression by targeting STAT3 (60). Li et al. showed that down-regulation of miRNA-98 was accompanied by up-regulation of IL-10 in tumor-associated macrophages of hepatocellular carcinoma (HCC), suggesting that IL-10 is a direct target of miRNA-98 (61). Li et al. showed that administration of liposomes carrying miRNA-98 could effectively reduce the frequency of B10 cells in tumor-bearing mice and inhibit experimental tumor growth, demonstrating that IL-10-producing B cells play an important role in tumor tolerance effect, while miRNA-98 up-regulation can suppress the expression of IL-10 in B cells, which helps suppress the body's tolerance to lung cancer (62). At the same time, miRNA-98 also plays an important role in myocarditis by inhibiting the expression of IL-10 in cardiac B cells (63). The miRNA-98 is involved in the transcriptional regulation of interleukin-10 in peripheral blood B cells of patients with airway allergy (64). Furthermore, inhibition of miRNA-98 reverses the ability of IL-10 expression in B cells (9). In addition, Rouas et al. showed that miR-27b-3p and miR-330-3p may be involved in post-transcriptional control of the immunosuppressive cytokines IL-10 and TGF-β (65). The miRNA-21 inhibits the differentiation of IL-10+ Breg cells and promotes autoimmunity by targeting the 3′ untranslated region of IL-10 mRNA (66). Other studies have shown that histone acetyltransferase p300 is involved in IL-4/miRNA-98/IL-10 axis in peripheral blood B cells of patients with airway hypersensitivity. Therefore, p300 inhibitors have therapeutic potential in the treatment of allergic diseases (64). Xiong et al. found that IL-10 negatively regulates miRNA-7025-5p, which down-regulates osteoblast differentiation. *In vivo* studies have shown that pre-injection of IL-10 results in increased bone formation, while injection of miRNA-7025-5p delays fracture healing. Therefore, IL-10 represents a promising therapeutic strategy to promote fracture healing (67). Li et al. found that miRNA-4492 was down-regulated in nasal polyps (NPs), while IL-10 was up-regulated in NPs, and the two were inversely related. This suggests that miRNA-4492/IL-10 axis involvement in the Jak/STAT signaling pathway may be a key mechanism for chronic sinusitis with NPs (68). Hatab et al. found that in patients with advanced liver cancer, 3 consecutive months of curcumin, piperine, and taurine combined treatment could reduce circulating levels of IL-10 and miRNA-21, and patients with high baseline levels of IL-10 and miRNA-21 had a worse overall survival rate. Therefore, changes in serum IL-10 and miRNA-21 expression may be prognostic biomarkers in the treatment of HCC (69). Finally, miRNA-27a enhances the antibacterial activity of macrophages and inhibits the expression of IL-10, thereby regulating the innate immune response (70). The relationship between miRNA and IL-10 levels in various diseases is summarized in Figure 3 and Table 2.

**Table 2:** Research on miRNA and IL-10 in diseases.

**MiRNA**	**Disease**	**Samples (*n*)**	**miRNA detection method**	**IL-10 detection method**	**miRNA expression**	**IL-10 level**	**References**
MiRNA-141	Breast cancer	Breast cancer (56), Healthy volunteer (6)	RT-qPCR	ELISA	DOWN	DOWN	(45)
MiRNA-146a/150	Elderly hip fracture	Elderly male Sprague Dawley rats (40), Young male SD rat (40)	RT-qPCR	ELISA	UP/DOWN	UP	(46)
MiRNA-10a	CAD	CAD (69), Control group (69)	Real-time PCR	ELISA	DOWN	DOWN	(47)
MiRNA-155	Lyme carditis	Mir155^−/−^ mice (3), IL-10^−/−^ Mir155^−/−^ (DKO) mice (3)	RT-qPCR	ELISA	UP	DOWN	(51)
MiRNA-19a	Allergen-related inflammation	Male C57BL/6 mice, miRNA-17-92fl/fl mice	RT-qPCR	ELISA	UP	DOWN	(54)
MiRNA-210	Preeclampsia	Late-onset preeclampsia (29), Normal uncomplicated pregnancies (27), Healthy and non-pregnant women (10)	RT-qPCR	ELISA	UP	DOWN	(55)
MiRNA−21-5p	HSP	HSP group (16), NHSP group (10)	Real-time PCR	FCM	DOWN	DOWN	(56)
rno-miRNA−211	ARDS	5-week-old male Wistar rats (12), Controls (12)	Microarray and Taqman assay	ELISA	UP	DOWN	(57)
MiRNA-223	RA	RA patients (22), Control group (19)	RT-qPCR	ELISA	UP	DOWN	(58)
rno- miRNA−30b-5p	Uveitis	Female Lewis rats (160)	Real-time PCR	ELISA	DOWN	UP	(59)
MiRNA-410	SLE	SLE patients (20), Healthy controls (20)	RT-qPCR	ELISA	DOWN	UP	(60)
MiRNA−98	HCC	Paired HCC (25), Adjacent normal tissues (25)	RT-qPCR	ELISA	DOWN	UP	(61)
MiRNA−98	Lung cancer	Male patients (5), Female patients (5)	RT-qPCR	ELISA	UP	DOWN	(62)
MiRNA-98	Myocarditis	Myocarditis heart tissue (3), Normal heart tissue (3)	RT-qPCR	ELISA	UP	DOWN	(63)
MiRNA-98	Allergic diseases	Asthma (10), Allergic rhinitis (14)	Real-time PCR	FCM	UP	DOWN	(64)
MiRNA-98	Atherosclerosis	Atherosclerosis (20), Healthy participants (20)	RT-qPCR	RT-qPCR	UP	DOWN	(9)

*RT-qPCR, reverse transcription-quantitative polymerase chain reaction; CAD, coronary artery disease; HSP, Henoch-Schonlein purpura; ARDS, acute respiratory distress syndrome; RA, rheumatoid arthritis; SLE, systemic lupus erythematosus; HCC, hepatocellular carcinoma; FCM, flow cytometry*.

## Regulatory miRNAs Targeting IL-10 May Provide New Therapeutic Targets for Many Diseases

IL-10 is a regulator of inflammation and immunosuppression. It regulates a variety of immune cells to limit and stop the inflammatory response, so it plays an important role in autoimmune diseases, inflammatory diseases, and cancer. Therefore, the regulatory effect of miRNA on IL-10 can be applied to many diseases that show changes in IL-10 levels, for the treatment or prognosis of diseases. Serum miRNA-146a and miRNA-150 can be used as biomarkers for diagnosis and prediction of ALI (46), and rno-miRNA-30b-5p is a new therapeutic target for uveitis (59). It is worth noting that miRNA-98 can be involved in the treatment of various diseases, such as lung cancer, liver cancer and other tumors, as well as myocarditis and allergic diseases (61, 62, 64, 71). In summary, regulatory miRNAs targeting IL-10 provide new therapeutic targets for many diseases, and also provide ideas for the development of new drugs for immune and inflammatory diseases in the future.

## LncRNA Associated With IL-10

LncRNA is defined as a long RNA transcript of more than 200 nucleotides that cannot be translated into a protein (72). Increasing amount of studies have shown that lncRNA plays a key role in the occurrence and development of cancer. Tang et al. found lncRNA actin filament-associated protein 1 antisense RNA 1 (AFAP1-AS1) was highly expressed in non-small cell lung cancer (NSCLC), accompanied by increased expression levels of IL-10 and interferon (IFN)-γ (73). Pei et al. found that lncRNA SNHG1 was increased in CD4+ TIL cells of breast cancer patients, while siRNA-SNHG1 can reduce the expression of Foxp3 and IL-10 (74). Ye et al. showed that lncRNA cox-2 siRNA can increase the expression levels of IL-10, Arg-1, and Fizz-1 in M2 macrophages (75). Zhou WY reported that the silencing of lnc-LINC00473 can reduce the expression of IL-10 in B-cell (76). In addition, Ma P. et al. found that overexpression of lnc-MALAT1 can increase IL-10 expression, thereby inhibiting neuronal apoptosis and promoting axon growth (77). Shi et al. found that increased expression of lncRNA-MALAT1 can decrease IL-10 expression, thereby up-regulating neuronal apoptosis and aggravating brain injury in rats with cerebral infarction (78). Li et al. found that lncRNA growth arrest specific transcript 5 (GAS5) was down-regulated in colorectal cancer (CRC), and subsequent functional experiments revealed that knockout of GAS5 promoted CRC cell proliferation and colony formation, while knockdown of GAS5 increases IL-10 expression and reduced CRC cell proliferation and colony formation (79).

## lncRNA and IL-10: A New Research Direction

Although lncRNA has become a research hotspot, we find that there is very little related research on lncRNA and IL-10. Therefore, there may be new discoveries related to lncRNA in the future research direction of IL-10.

## Summary

IL-10 is a highly potent cytokine produced by a variety of cells. It regulates cell growth and differentiation, participates in inflammatory reactions, and immune responses, and is currently recognized as inflammation and immunosuppressive factor. It plays an important role in autoimmune diseases and inflammatory diseases. Therefore, it is also an important means of treating related diseases. Almost all lymphocytes in the body can synthesize IL-10, including mononuclear macrophages, DCs, B cells, etc. Increased IL-10 expression can cause immunosuppression and SLE, RA, asthma, gastric cancer, and other diseases. Also, IL-10 plays a decisive role in the development of immune paralysis, the development of temporary immunodeficiency after trauma, major surgery, burns, shock, and high-risk bacterial/fungal infections.

There are many studies on the epigenetics of IL-10. In different diseases, the DNA methylation modification of IL-10 gene locus can affect the occurrence and development of the disease. In addition, miRNA-98 is an important miRNA, which is associated with IL-10 in various diseases, such as HCC, Lung cancer, Myocarditis, etc. (61, 62, 71). These studies have shown that the epigenetic mechanism of IL-10 help us to have a deeper understanding of the occurrence and progression of the disease. However, the current IL-10 related epigenetics research has not yet involved the diagnosis and clinical treatment of diseases. A recent review (3) describes the epigenetic mechanism controlling IL-10 expression, with an emphasis on some transcription factors in the construction of chromatin landscape in IL-10 induced T helper cell (3). Here, we present the epigenetic mechanisms controlling IL-10 expression in various diseases, and we also review the regulation of IL-10 gene expression in terms of DNA methylation, histone modification, miRNA, and lncRNA.

Epigenetics plays an important role in the regulation of IL-10. Changes in DNA methylation levels, histone acetylation or deacetylation affect IL-10 expression. At the same time, miRNA plays a regulatory role in the late IL-10 transcription, and the mechanism of circRNA and IL-10 related to immune inflammation is still unclear, and it is still a blank, providing ideas and directions for future research. The mechanisms of IL-10 production and regulation are very complicated, and we need to continue to research and discover in the future.

## Author Contributions

SD and ZZ contributed to the conception, design and final approval of the submitted version, and writing the paper. GH and TG contributed to interpretation of data. TH, MZ, and YZ contributed to the completion of figures and tables. All authors have read and approved the final manuscript.

## Conflict of Interest

The authors declare that the research was conducted in the absence of any commercial or financial relationships that could be construed as a potential conflict of interest.
